# The S100A4 D10V polymorphism is related to cell migration ability but not drug resistance in gastric cancer cells

**DOI:** 10.3892/or.2014.3540

**Published:** 2014-10-13

**Authors:** TEIN-MING YUAN, RUEI-YUE LIANG, NAI-WAN HSIAO, SHOW-MEI CHUANG

**Affiliations:** 1 Institute of Biomedical Sciences, National Chung Hsing University, Taichung 40227, Taiwan, R.O.C.; 2 Department of Surgery, Feng-Yuan Hospital, Ministry of Health and Welfare, Taichung 42055, Taiwan, R.O.C.; 3 Institute of Biotechnology, National Changhua University of Education, Changhua 50007, Taiwan, R.O.C.

**Keywords:** S100A4, gastric cancer, polymorphism, migration

## Abstract

Upregulation of the metastasis-promoting S100A4 protein has been linked to tumor migration and invasion, and clinical studies have demonstrated that significant expression of S100A4 in primary tumors is indicative of poor prognosis. However, the involvement of S100A4 in the drug responsiveness of gastric cancer remains unclear. In the present study, we used gastric cancer cell lines as a model to investigate the involvement of S100A4 in drug responsiveness. We overexpressed S100A4 in AGS and SCM-1 cells, which are characterized by relatively low-level expression of endogenous S100A4, and found that this significantly enhanced cell migration but did not affect cell survival in the presence of six common anticancer drugs. Moreover, *in vitro* cell proliferation was unchanged. Using RNA interference, we suppressed S100A4 expression in MKN-45 and TMK-1 cells (which are characterized by high-level expression of endogenous S100A4), and found that knockdown of S100A4 markedly attenuated cell motility but did not affect cell survival in the presence of six common anticancer drugs. Further study revealed that a single nucleotide polymorphism (SNP) of S100A4 (rs1803245; c.29A>T), which substitutes an Asp residue with Val (D10V), is localized within the conserved binding surface for Annexin II. Cells overexpressing S100A4^D10V^ showed a significant reduction in cell migration ability, but no change in cell survival, upon anticancer drug treatment. Taken together, our novel results indicate that the expression level of S100A4 does not significantly affect cell survival following anticancer drug treatment. Thus, depending on the cell context, the metastasis-promoting effects of S100A4 may not be positively correlated with anticancer drug resistance in the clinic.

## Introduction

The S100 proteins comprise a family of more than 25 different members (1). Found exclusively in vertebrates, these proteins are involved in activating specific biochemical pathways to regulate various cellular functions, including proliferation, survival, differentiation and motility (2). The S100 protein family has attracted increasing attention in the field of cancer research; in particular, S100A4 reportedly contributes to various aspects of tumor progression, including cell motility, metastasis and angiogenesis (3–5). S100A4 was first isolated as the product of a gene that is differentially expressed in highly metastatic mouse mammary adenocarcinoma cells (6). It has since been shown to be specifically upregulated in aggressive and advanced metastatic tumors relative to non-invasive, non-metastatic tumors (7,8). Elevated expression of S100A4 has been found in numerous cancer types; its expression in non-metastatic cell lines was shown to trigger a more metastatic phenotype (9,7), whereas decreased S100A4 expression was associated with a lower metastatic capacity (10,11). Furthermore, transgenic animal studies have established positive associations between S100A4 and both metastasis and tumor development (12–15). Clinical studies have convincingly demonstrated that significant expression of S100A4 in primary tumors is indicative of poor prognosis (16–18), and that S100A4 may be a useful marker for predicting the development, progression and metastasis of human gastric cancer (19).

Despite these findings, however, we do not yet fully understand the exact mechanisms through which S100A4 executes its pro-metastatic functions. Intracellular S100A4 has been shown to interact with non-muscle myosin IIA at the leading edge of migrating cells, which may promote cell migration (20). In addition, S100A4 may modulate the expression levels of MMP9 and MMP13, thereby regulating invasion and metastasis in human prostate and breast cancer cells (21,22), respectively. In esophageal squamous cell carcinoma, the ability of S100A4 to promote tumor invasion and metastasis is associated with the upregulation of MMP2 and the down-regulation of E-cadherin (23). Moreover, Lo *et al* showed that S100A4 induced epithelial-mesenchymal transition (EMT) to maintain the stemness of cancer cells and the tumorigenic properties of head and neck cancers (24).

In addition to acting intracellularly, some of the S100 proteins demonstrate extracellular activity by acting as chemo-attractants. S100A4 can be secreted, and several lines of evidence suggest that it can induce cytokine networks, such as those mediated by the inflammatory cytokines IL8, CCL2 and SAA, thereby enabling tumor cells to engage with angiogenic and inflammatory stromal cells (25,26). In this regard, S100A4 is believed to have potential as a highly prognostic molecular biomarker for metastatic potential, as already shown for breast, colorectal, gallbladder, pancreatic as well as other types of cancer (3,18).

However, although data indicate that high-level expression of S100A4 is associated with increased metastatic capacity, we are only just beginning to unravel the potential roles of this protein in chemoresistance. Moderate S100A4 overexpression was found in a doxorubicin-resistant colon cancer cell line compared to doxorubicin-sensitive cells (27), whereas S100A4 knockdown was associated with upregulation of BNIP3, increased sensitivity of pancreatic ductal adenocarcinoma cell lines to gemcitabine treatment, and enhanced apoptosis (28). Furthermore, S100A4 mRNA and protein levels were found to be upregulated in methotrexate (MTX)-resistant cancer cells and to contribute to MTX resistance (29). Other S100 family proteins have also been demonstrated to contribute to chemoresistance (30,27). Despite these previous findings, however, the involvement of S100A4 in the drug responsiveness of gastric cancer remains less well understood.

Considering the upregulation of S100A4 in metastatic tumors and the literature correlating its expression with poor prognosis, we investigated whether S100A4 may mediate chemotherapeutic resistance in gastric cancer. Here, we reported that ectopic expression of S100A4 did not promote anticancer drug resistance in gastric cancer cells, and S100A4 knockdown had little effect on the survival of drug-treated cells. These data strongly suggest that, depending on the cell context, the metastasis-promoting effect of S100A4 may not be positively correlated with anticancer drug resistance in the clinic.

## Materials and methods

### Cell culture

The human gastric carcinoma cell lines, AGS, TMC-1, SNU-1, TMK-1, SCM-1, MKN-45, and KATO III, were cultured in RPMI-1640 (Invitrogen, Carlsbad, CA, USA) supplemented with 10% fetal bovine serum (FBS), sodium bicarbonate (2%, w/v), L-glutamine (0.29 mg/ml), penicillin (100 U/ml), and streptomycin (100 μg/ml) (Invitrogen) at 37°C in a humidified 5% CO_2_ incubator.

### Antibodies and chemicals

Specific antibodies against S100A4, ribophorin II (RPN2) and β-actin were obtained from Santa Cruz Biotechnology (Santa Cruz, CA, USA). Anti-PARP and anti-caspase-3 were obtained from Cell Signaling Technology (Beverly, MA, USA). Anti-Myc was purchased from Millipore (Millipore Corporation, Bedford, MA, USA). Cisplatin was purchased from Sigma (St. Louis, MO, USA).

### MTS assays

Cells (5×10^3^) were seeded in 96-well culture plates, incubated overnight at 37°C in medium containing 10% FBS, and then treated with the indicated concentrations of anticancer drugs for 48 h. Cell viability was determined using an MTS colorimetric assay (CellTiter 96^®^ cell proliferation assay kit; Promega, Madison, WI, USA) as described by the manufacturer. All experiments were performed at least in triplicate, on three separate occasions. A dose-response curve was plotted, and the drug concentration that decreased color development by 50% (i.e., the IC_50_ value) was calculated for each drug. The data are presented as means ± SDs.

### RNA interference

For small-interfering RNA (siRNA) knockdown of S100A4, ON-TARGET plus SMART pool siRNAs against S100A4 were purchased from Dharmacon Research (Lafayette, CO, USA). Non-targeting siRNA duplexes were used as negative controls (Dharmacon Research). Cells were transfected with siRNA using Lipofectamine RNAiMAX (Invitrogen) and incubated in glucose-free Opti-MEM (Invitrogen) according to the manufacturer’s recommendations.

### Western blot analysis

Cell extracts were prepared in lysis buffer (50 mM HEPES, pH 7.5, 150 mM NaCl, 5 mM EDTA, 1% Triton X-100, 50 mM NaF, 1 mM Na_3_VO_4_, 10% glycerol, and a protease inhibitor cocktail). Equal amounts of proteins were separated by sodium dodecyl sulfate-polyacrylamide gel electrophoresis (SDS-PAGE) and transferred to polyvinylidene difluoride (PVDF) membranes (Millipore, Billerica, MA, USA). The membranes were blocked, washed, probed with the indicated primary antibodies, washed again, and incubated with horseradish peroxidase-conjugated secondary antibodies for 1 h. Finally, the blots were washed, and then developed using enhanced chemiluminescence (ECL) reagents (Millipore) according to the manufacturer’s protocol.

### Reverse transcription-polymerase chain reaction (RT-PCR) analysis

RNA was isolated from cultured cells using the TRIzol reagent (Invitrogen) according to the manufacturer’s instructions, and cDNA was synthesized from 2 μg of total RNA by reverse transcription using the ImProm-II Reverse Transcriptase kit (Promega) with oligo(dT)_12–18_ primers. The resulting cDNA was used for subsequent PCR using specific PCR primers for S100A4 (forward, 5′-ATGGCGTGCCCTCTGGAG-3′ and reverse, 5′-TTTCTTCCTGGGCTGC-3′).

### Site-directed mutagenesis

The full-length human S100A4 cDNA was inserted into pcDNA3.1 (Invitrogen) to express the S100A4-Myc recombinant protein. Asp-10 was substituted with a valine residue using a QuikChange II Site-Directed Mutagenesis kit (Stratagene, La Jolla, CA, USA) using specific DNA oligonucleotides (D10V forward, 5′-GAGAAGGCCCTGGTTGTGATGGTGTCC-3′ and D10V reverse, 5′-GGACACCATCACAACCAGGGCCTTCTC-3′).

### Real-time cell analysis (RTCA) system

For continuous monitoring of cell migration, cells (1×10^4^ cells/well) suspended in serum-free medium were seeded into the upper compartment of CIM-plates 16 (Roche, Mannheim, Germany). The lower compartment was then filled with medium containing 10% FBS and incubated in the RTCA station (xCELLigence System, Roche). Cell migration was monitored for 24 h with impedance measured every 15 min. Cell impedance was represented as cell index (CI) = (Z_i_-Z_0_) [Ohm]/15[Ohm], where Z_0_ was the background resistance and Z_i_ was the resistance at a given time-point. A normalized cell index was determined as the cell index at a given time-point (CI_ti_) divided by the cell index at the normalization time-point (CI_nml_time_).

### Cell migration assay

The *in vitro* cell migration assay was performed in Transwell chambers (Millipore), using 8.0-μm pore-size filters according to the manufacturer’s recommendations. Briefly, 1×10^5^ cells were suspended in serum-free DMEM and seeded to the upper compartment of a Transwell insert, while the lower compartment was loaded with a 24-well dish containing medium supplemented with 10% FBS. After incubation at 37°C in 5% CO_2_ for 18 h, a cotton swab was used to remove the non-migrated cells from the upper surface of the membrane. The cells that had migrated through the membrane and adhered to the lower surface were fixed with methanol and then stained with crystal violet (1% crystal violet in 75% ethanol). The cells were examined under a microscope and counted. Experiments were performed in triplicate, and the results were calculated by averaging the total number of cells from three membranes.

### Statistical analyses

All experiments were performed in triplicate. The significances of between-group differences were determined using the Student’s t-test. A P-value <0.05 was considered to indicate a statistically significant difference.

## Results

### S100A4 expression and cell migration

**Figure 1:**
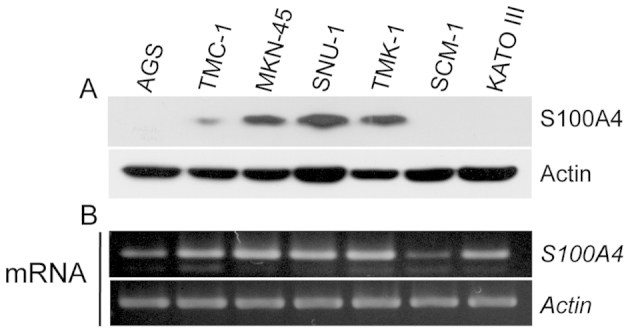
S100A4 expression in seven gastric cancer cell lines. (A) Cell extracts were prepared from exponential growing cells. After determination of protein concentration, equal amounts of extracts were conducted to SDS-PAGE followed by western blot analysis using antibody specific to S100A4. (B) Total RNA was isolated from cultured cells as described in Materials and methods. The RNA levels of S100A4 in each cell line were determined.

**Figure 2:**
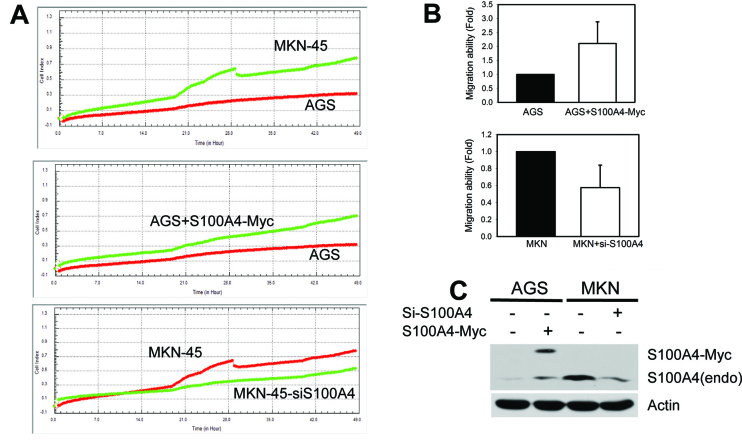
S100A4 expression is correlated with cell migration. (A) Exponential growing cells were transfected with S100A4-Myc (for AGS) or siRNA against S100A4 (for MKN-45) for 24 h. The cell migration ability was assayed by real-time cell analysis (RTCA) system, as described in Materials and methods. The fold of migrated cells was further calculated. (B) Values (means ± SDs) are from at least three independent experiments. (C) Exponential growing cells were transfected with S100A4-Myc (AGS) or siRNA against S100A4 (MKN-45) for 24 h and the protein levels of S100A4 and β-actin were determined by immunoblot analysis using specific antibodies.

First, we examined the expression levels of S100A4 in seven gastric cancer cell lines. S100A4 was found to be highly expressed in MKN-45, SNU-1 and TMK-1 cells at both the protein and mRNA levels, whereas AGS and SCM-1 cells exhibited much lower expression of S100A4 at the protein and mRNA levels (Fig. 1). We therefore used AGS, MKN-45, TMK-1, and SCM-1 cells in our subsequent studies. To examine the functional significance of S100A4 in the cell migration of these gastric cancer cell lines, we used the RTCA system, which is a label-free, real-time automated continuous-monitoring platform that assesses cell migration by measuring changes in the electrical impedance at the electrode/cell interface. As shown in Fig. 2, higher expression levels of endogenous S100A4 were significantly associated with the higher migration ability of MKN-45 cells compared to AGS cells. Moreover, the migration ability of AGS cells was markedly enhanced by ectopic expression of S100A-Myc, whereas siRNA-mediated knockdown of endogenous S100A4 expression attenuated the cell migration of MKN-45 cells (Fig. 2A–C). Consistent with the results from previous reports, our data confirmed that S100A4 plays a critical role in the cell migration of gastric cancer cell lines.

### S100A4 expression and drug responsiveness

**Figure 3:**
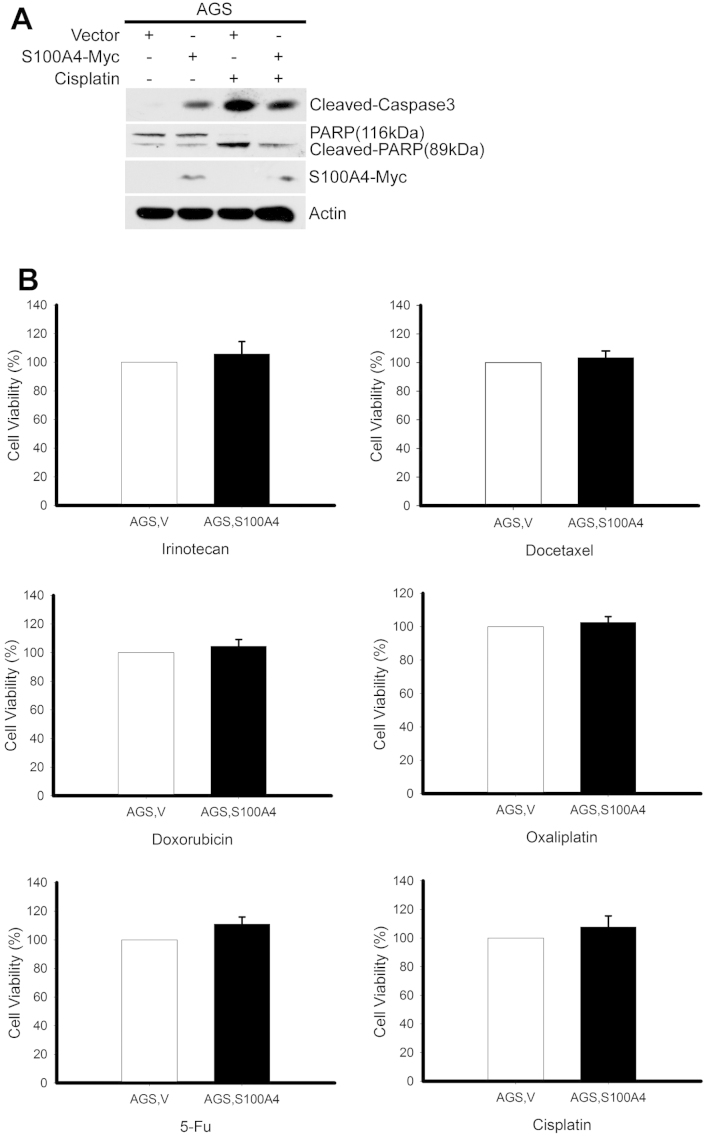
Ectopic S100A4 expression does not affect anticancer drug-induced cytotoxicity in AGS cells. (A) Exponential growing cells were transfected with S100A4-Myc for 24 h and the protein levels of S100A4, cleavage products of caspase 3 and PARP were determined. (B) Exponential growing cells were transfected with S100A4-Myc for 24 h and then treated with 7.4 μM irinotecan, 2.4 nM docetaxel, 46 nM doxorubicin, 10.6 μM oxaliplatin, 28.8 μM 5-fluorouracil (5-FU) and 4 μg/ml cisplatin for 48 h. Cell viability was determined by MTS assay. The values are from at least three independent experiments.

**Figure 4:**
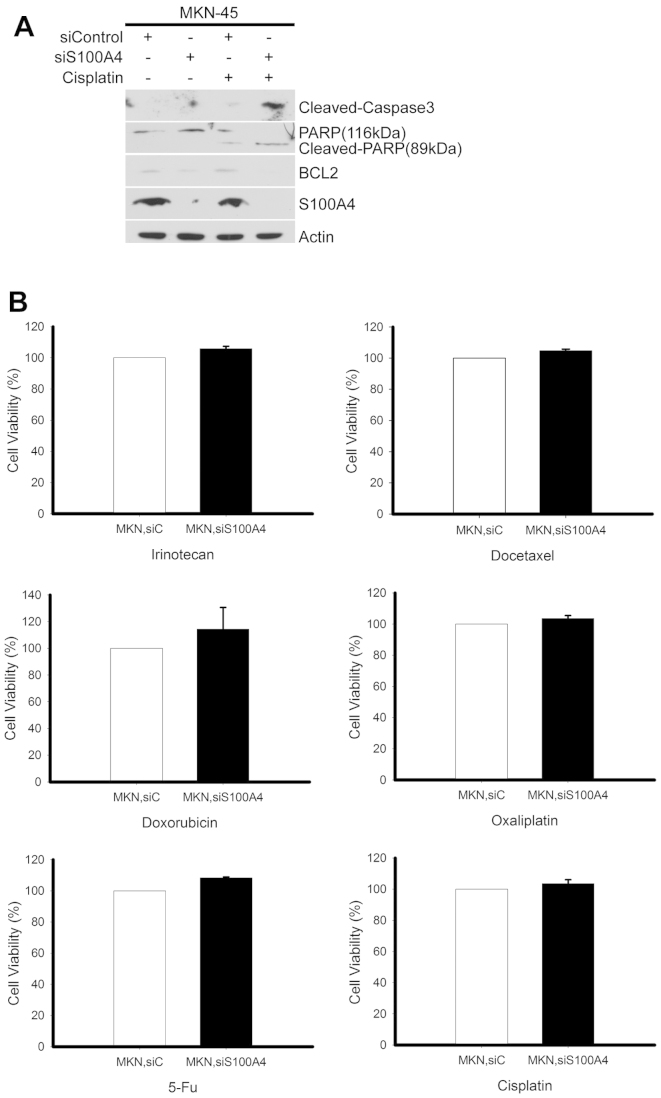
S100A4 silencing does not increase drug-induced cytotoxicity in MKN-45 cells. (A) Exponential growing cells were transfected with siRNA against S100A4 for 24 h and the protein levels of S100A4, cleavage products of caspase 3, PARP and BCL2 were determined. (B) Exponential growing cells were transfected with siRNA against S100A4 for 24 h and then treated with 11.6 μM irinotecan, 1.9 nM docetaxel, 103.6 nM doxorubicin, 14 μM oxaliplatin, 10.3 μM 5-fluorouracil (5-FU) and 2.5 μg/ml cisplatin for 48 h as described in Figure 3. Cell viability was determined by MTS assay. The values are from at least three independent experiments.

**Figure 5:**
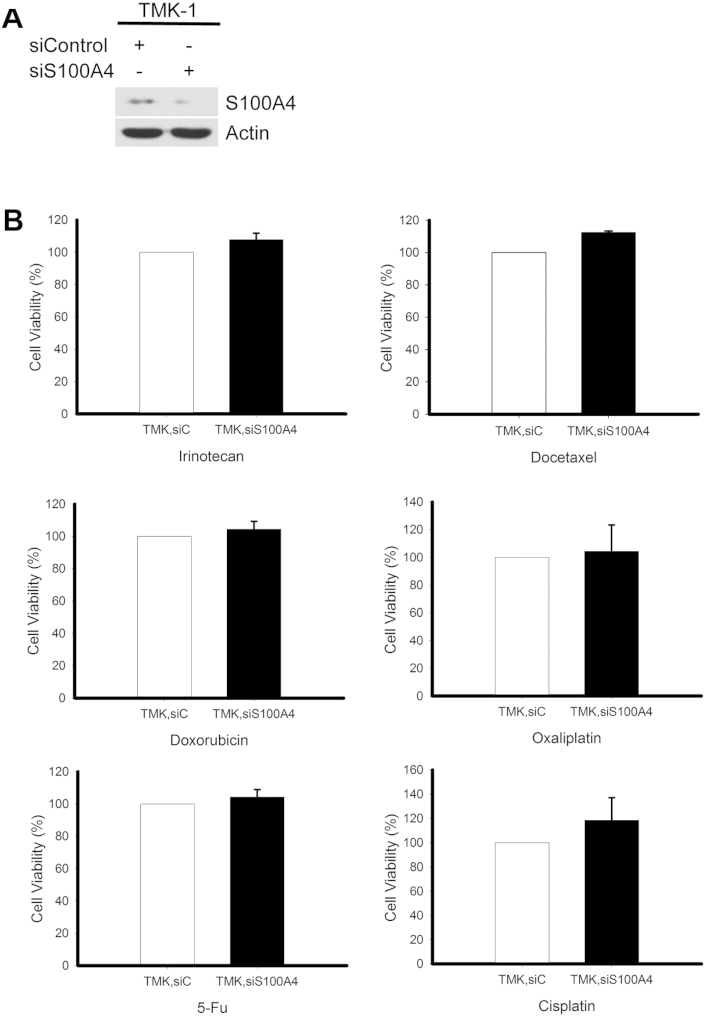
S100A4 silencing does not increase drug-induced cytotoxicity in TMK-1 cells. (A) siRNA transfection and protein determination were performed as described in Fig. 3. (B) After transfection, cells were treated with 11.6 μM irinotecan, 0.9 nM docetaxel, 124.4 nM doxorubicin, 22.6 μM oxaliplatin, 140.5 μM 5-fluorouracil (5-FU) and 1.7 μg/ml cisplatin for 48 h. Cell viability was determined by MTS assay. The values are from at least three independent experiments.

To investigate the role of S100A4 in anticancer drug resistance, AGS cells expressing exogenous S100A4-Myc were exposed to the half-maximal inhibitory concentrations (IC_50_) of six conventional anticancer drugs for 48 h. Immunoblot analysis of apoptosis-related protein markers revealed that overexpression of S100A4 slightly decreased the cisplatin-induced cleavage of caspase-3 and poly(ADP-ribose) polymerase (PARP) in AGS cells (Fig. 3A). MTS-based cell viability analyses showed that overexpression of S100A4 slightly (but not significantly) increased cell viability relative to empty-vector controls in AGS cells treated with any of the tested drugs (Fig. 3B). Next, MKN-45 cells were subjected to siRNA-mediated knockdown of S100A4 (siS100A4) for 24 h and then challenged with anticancer drugs for 48 h. S100A4 knockdown slightly increased the cisplatin-induced cleavage of caspase-3 and PARP, and decreased BCL2 protein levels (Fig. 4A). However, our MTS-based analysis of cell viability indicated that knockdown of S100A4 did not have any discernible effect on cell survival (Fig. 4B). A similar pattern of drug responsiveness was obtained in TMK-1 cells, wherein significant knockdown of S100A4 (Fig. 5A) did not alter the resistance to various anticancer drugs (Fig. 5B).

### The S100A4 D10V polymorphism affects the cell migration ability of gastric cancer cells

**Figure 6:**
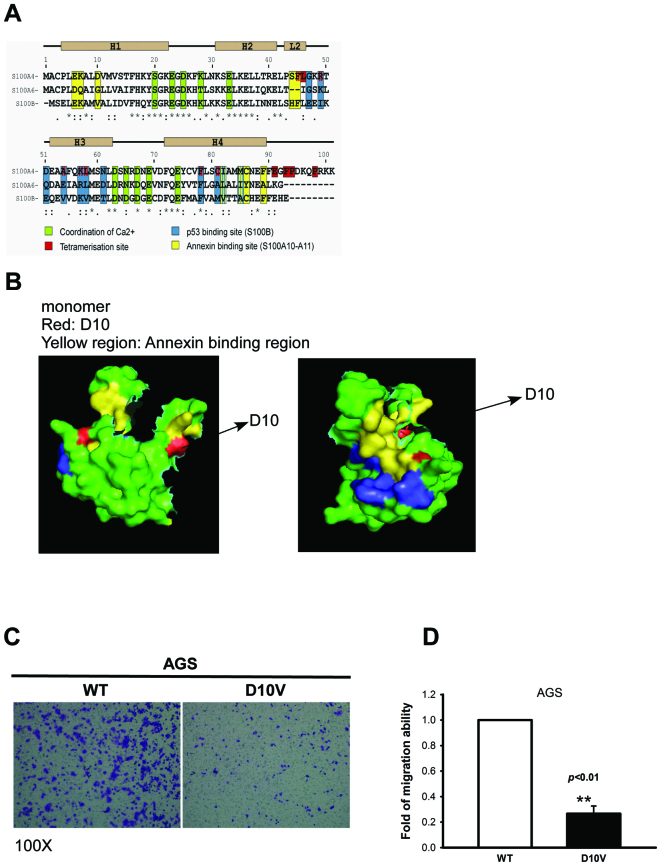
SNP c.29A>T affects cell migration ability. (A) Nucleotide sequence comparison among S100 proteins. Yellow regions were determined as the Annexin binding sites. (B) The 3D structure modeling of S100A4. The arrow indicates the location of D10 amino acid. (C) AGS was transfected with wild-type or S100A4^D10V^ mutant for 24 h and then the cell migration was determined by Transwell as described in Materials and methods. (D) The fold of migrated cells was further calculated. Values (means ± SDs) are from at least three independent experiments. ^**^P<0.01 as compared with wild-type.

Numerous proteins have been identified as potentially interacting with S100 proteins, including Annexin II. (18,31) We selected nonsynonymous single-nucleotide polymorphisms (SNPs) in S100A4 from dbSNP, and examined whether they alter the biological function of the protein. We identified an SNP of S100A4 (NM_002961.2: c.29A>T, rs1803245) that resulted in the substitution of an Asp residue with a Val residue (NP002952.1:p.Asp10Val), and further found that it localizes within a sequence that is conserved among the members of the S100A protein family (Fig. 6A). Computer modeling of the S100A4 protein structure showed that the D10 residue is localized within the binding surface for Annexin II (Fig. 6B). To examine the potential significance of this SNP in cancer cell migration and anticancer drug resistance, we next examined whether cancer cells expressing this S100A4 protein variant showed changes in their metastasis-related properties. AGS cells were transfected with wild-type S100A4^D10^ (A allele) or S100A4^D10V^ (T allele), and cell motility was assessed by an *in vitro* cell migration assay. Compared to cells expressing S100A4^D10^, those expressing S100A4^D10V^ showed significantly fewer instances of traversing the membrane (P<0.01) (Fig. 6C and D). This suggests that S100A4^D10V^ is negatively correlated with cancer cell motility compared to the wild-type.

### The S100A4 D10V polymorphism does not affect anticancer drug responsiveness in gastric cancer cells

**Figure 7:**
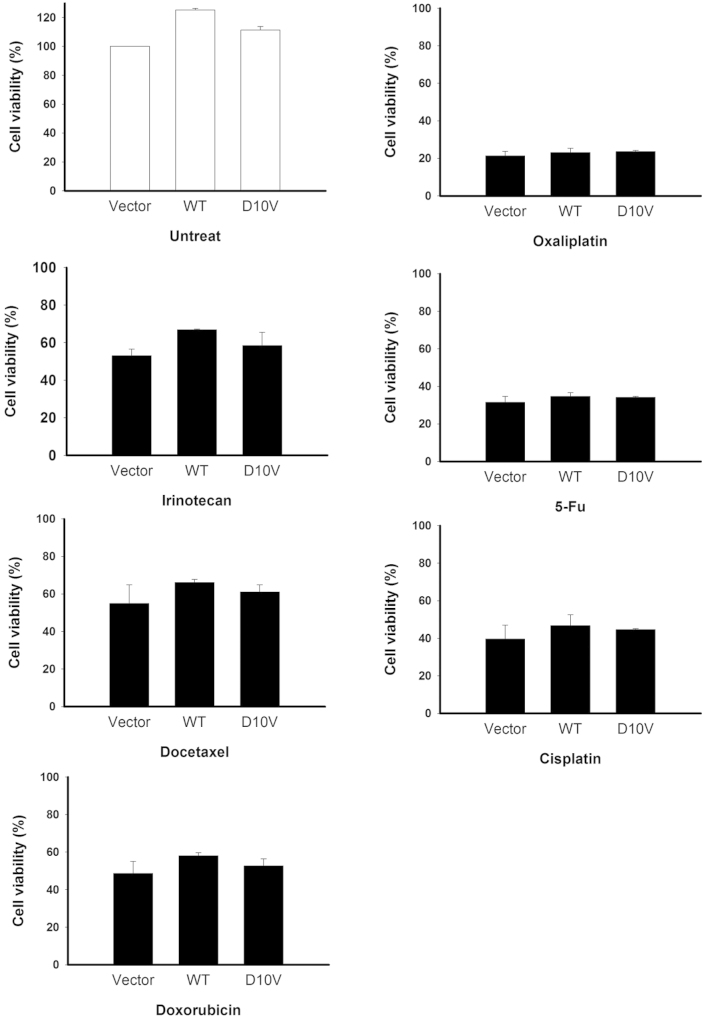
S100A4^D10V^ does not have any effect on anticancer drug-induced cytotoxicity in AGS cells. Cells were overexpressed with wild-type or S100A4^D10V^ for 24 h and then exposed to anticancer drugs for 48 h as described in Fig 3. Cell viability was determined by MTS assay. The values are from at least three independent experiments.

**Figure 8:**
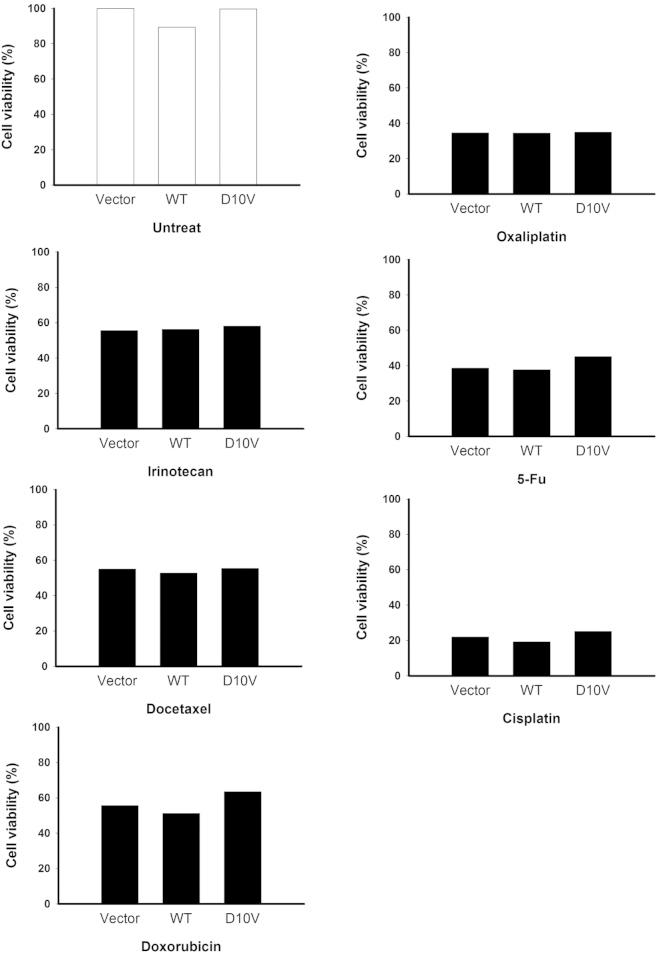
S100A4^D10V^ does not have any effect on anticancer drug-induced cytotoxicity in SCM-1 cells. After transfection, cells were exposed to 8.7 μM irinotecan, 1.4 nM docetaxel, 62.1 nM doxorubicin, 17.5 μM oxaliplatin, 91 μM 5-fluorouracil (FU) and 1.3 μg/ml cisplatin for 48 h. Cell viability was determined by MTS assay.

The drug responsiveness of cells expressing S100A4^D10V^ was further investigated. AGS cells overexpressing S100A4^D10V^ were individually treated with the six tested anticancer drugs for 48 h, and cell viability was determined. As shown in Fig. 7, the D10V substitution was not found to correlate with drug responsiveness. Similar results were obtained in SCM-1 cells expressing S100A4^D10V^ (Fig. 8). Overexpression of both S100A4 versions did not have any discernible effect on cell growth in the absence of drugs (Figs. 7 and 8), with the exception of a slight enhancement of viability among AGS cells (Fig. 7). Taken together, our results suggest that S100A4 contributes to cancer cell migration but not the resistance to anticancer drugs.

### Dual knockdown of S100A4 and RPN2 does not affect drug responsiveness

**Figure 9:**
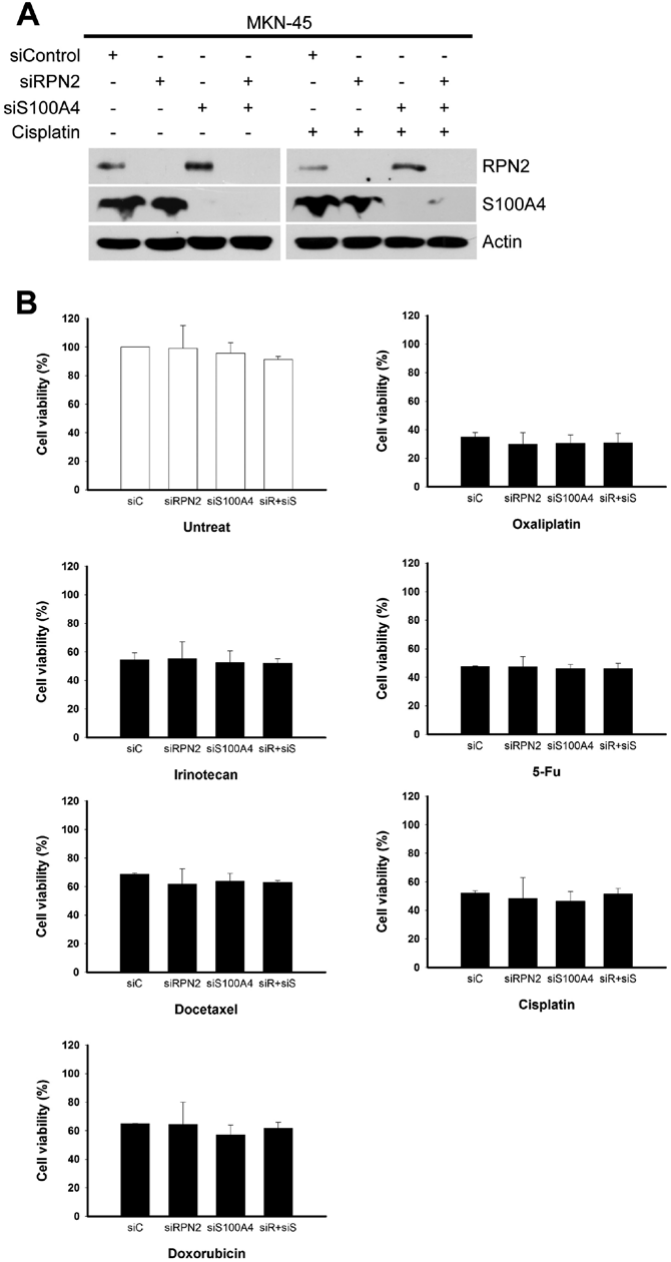
S100A4 and RPN2 co-knockdown does not have discernible effects on cell survival. (A) MKN-45 cells were transfected with siRNA against S100A4 and RPN2. The protein levels of S100A4, RPN2 and β-actin were determined by western blot analysis. (B) MKN-45 cells depleted with S100A4 and RPN2 expression were administered with drugs for 48 h as described in Fig 4. Cell viability was determined by MTS assay. The values are from at least three independent experiments.

Ribophorin II (RPN2) is a prognostic marker and has been shown to contribute to resistance against chemotherapeutic agents in human breast tumors and animal models of breast cancer (32–36). Therefore, we tested whether RPN2 depletion could have a synergistic effect in S100A4-knockdown gastric cancer cells. MKN-45 cells were subjected to simultaneous siRNA-mediated depletion of RPN2 and S100A4 for 24 h, and individually challenged with the six tested anticancer drugs for another 48 h. MTS-based assays of cell survival revealed that there was no change in cell viability among cultures subjected to RPN2 knockdown, S100A4 knockdown, or dual knockdown of RPN2 plus S100A4, regardless of drug treatment (Fig. 9).

## Discussion

Researchers have investigated S100A4 for its involvement in multiple aspects of tumor progression, such as proliferation, apoptosis, cell motility, extracellular matrix remodeling and angiogenesis. Recent studies have shown that elevated S100A4 protein levels are positively correlated with various human tumors and are associated with poor prognosis in human gastric, colorectal, pancreatic, thyroid, breast, lung, prostate and renal cell cancer (18). However, the potential involvement of S100A4 in chemoresistance is not yet fully understood. To further explore the potential role of S100A4 in the drug resistance of gastric cancers, we herein investigated the correlation of S100A4 expression levels with the survival of gastric cancer cell lines exposed to anticancer drug treatment. We found that siRNA-mediated knockdown of S100A4 in gastric cancer cell lines significantly reduced cell migration but did not alter cell viability following administration of the tested drugs. Moreover, migration assays showed that cells overexpressing the S100A4^D10V^ variant had significantly fewer instances of traversing the membrane, suggesting that the D10V (rs1803245) polymorphism may interfere with the interaction between S100A4 and Annexin II. Taken together, our data indicate that there is no significant correlation between S100A4 status and chemoresistance in the tested gastric cancer cell lines.

Several recent studies have evaluated S100A4 as a therapeutic target in preclinical models (37), and some have found that S100A4 is negatively correlated with cell growth in tumor cells. For example, the metastatic phenotype of human osteosarcoma cells was significantly inhibited by a ribozyme directed against the S100A4 gene transcript, but no effect was observed on cell proliferation or tumorigenicity *in vitro* and *in vivo* (14). A whole-human-genome microarray analysis identified S100A4 as being differentially expressed between MTX-sensitive and -resistant cells (29), showing overexpression in five of the seven tested MTX-resistant cell lines. Knockdown of S100A4 increased the sensitivity of HT29 cells toward MTX, but knockdown of S100A4 had no effect on cell viability in HT29 MTX-resistant cells, suggesting that other proteins may coordinately fulfill the drug resistance (29). Conversely, S100A4 depletion was found to suppress cell proliferation and invasiveness in pancreatic cancer cell lines characterized by high-level expression of endogenous S100A4 (38). Moreover, forced expression of S100A4 markedly accelerated the cell migration of pancreatic cancer cell lines with relatively low-level endogenous expression of S100A4, but did not affect their cell growth or invasion ability (5).

Based on the above somewhat contradictory findings, we herein analyzed the potential involvement of S100A4 in the cell motility and proliferation of gastric cancer cell lines. We failed to obtain convincing evidence supporting the involvement of S100A4 in the survival of the tested cell lines. The complex phenomenon of tumor chemoresistance likely requires both gains and losses of various functions, enabling escape from the cytotoxicity induced by chemotherapeutic agents in tumor cells. Our present results suggest that in spite of S100A4, the acquisition of cell survival and growth ability in response to chemotherapy may result from the integration of other factors, at least among the tested gastric cancer cell lines.

Although the clinical correlation between S100A4 expression and tumor metastasis is more conclusive, future studies are warranted to define the relevant interactions between S100A4 and its binding partners. Several downstream targets and interaction partners of S100A4 are involved in S100A4-mediated tumor migration and invasion (18), and S100A4 reportedly interacts with multiple molecular proteins involved in the metastatic processes of cytoskeletal rearrangement and cell motility, including F-actin, tropomyosin and the heavy chain of nonmuscle myosin II (18). By surveying an SNP database and performing computer modeling, for the first time, we identified an SNP in S100A4 (NM_002961.2: c.29A>T, rs1803245); encoding a valine substitution at D10, it is localized within the potential binding surface to Annexin II. Cell migration assays showed that the D10V substitution reduced the migration ability of the tested cell lines, as compared to cells overexpressing wild-type S100A4. Future studies are required to examine whether the interaction between S100A4 and Annexin II has functional effects, perhaps contributing to the metastatic phenotype.

In conclusion, it is likely that multiple dysregulated pathways synergistically contribute to chemoresistance in cancer cells. Here, we report that S100A4 appears to have little effect on drug responsiveness in various gastric cancer cell lines, suggesting that we should identify other relevant factors in order to improve our therapeutic strategies against gastric cancer.
